# Gestational diabetes mellitus subtypes according to oral glucose tolerance test and pregnancy outcomes

**DOI:** 10.1007/s12020-025-04329-1

**Published:** 2025-06-26

**Authors:** Kleoniki I. Athanasiadou, Stavroula A. Paschou, Georgios Markozannes, Vasiliki Vasileiou, Fotini Kanouta, Marina Mitropoulou, Panagiotis Antsaklis, Mariana Theodora, Theodora Psaltopoulou, George Daskalakis, Dimitrios G. Goulis, Eleni Anastasiou

**Affiliations:** 1 Endocrine Unit and Diabetes Centre, Department of Clinical Therapeutics, Alexandra Hospital, School of Medicine, National and Kapodistrian University of Athens, Athens, Greece; 2 Department of Hygiene and Epidemiology, School of Medicine, University of Ioannina, University Campus, Ioannina, Greece; 3 Department of Endocrinology and Diabetes Centre, Alexandra Hospital, Athens, Greece; 4 1st Department of Obstetrics and Gynecology, Alexandra Hospital, National and Kapodistrian University of Athens, Athens, Greece; 5 Unit of Reproductive Endocrinology, 1st Department of Obstetrics and Gynaecology, Medical School, Aristotle University of Thessaloniki, Thessaloniki, Greece

**Keywords:** GDM subtypes, Gestational diabetes mellitus, Heterogeneity, OGTT, Pregnancy

## Abstract

**Purpose:**

To determine whether the abnormal glucose concentrations at various oral glucose tolerance test (OGTT) time points are associated with adverse perinatal outcomes in pregnancies complicated by gestational diabetes mellitus (GDM).

**Methods:**

A retrospective study included 257 pregnant women with GDM (IADPSG criteria) who delivered between 2020–2023 at a tertiary hospital. Women were classified based on their OGTT results: isolated fasting hyperglycemia (group A), isolated post-load hyperglycemia (group B), and combined hyperglycemia (group C). Multivariable linear and logistic regression analyses were performed.

**Results:**

Most women had fasting hyperglycemia (54.1%), followed by isolated post-load hyperglycemia (29.2%), and combined hyperglycemia (16.7%). In the univariate analysis, women in Groups A and C had higher BMI before pregnancy (29.0 [7.6] kg/m^2^ and 30.6 [9.3] kg/m^2^, respectively) compared with women in Group B (26.3 [6.2] kg/m^2^). Groups A and C had a higher prevalence of insulin use compared with Group B (81.3 and 88.4% vs. 49.3%, *p* < 0.001). Their neonates had higher birth weights (3221 ± 525 g and 3208 ± 512 g vs. 3030 ± 591 g, *p* = 0.039) and higher rates of large-for-gestational-age (11.5 and 16.3% vs. 2.7%, *p* = 0.032). However, the multivariable analyses did not show significant differences among the groups.

**Conclusion:**

The GDM subtypes identified through the OGTT were related to distinct metabolic phenotypes and pregnancy outcomes, indicating the presence of heterogeneity in GDM. Future studies are required to confirm these findings and explore whether the OGTT could be used to guide individualized GDM treatment.

## Introduction

The oral glucose tolerance test (OGTT) is the gold standard for diagnosing gestational diabetes mellitus (GDM). The publication of the Hyperglycemia and Adverse Pregnancy Outcomes (HAPO) study results in 2008 radically changed the diagnostic approach of GDM. The study showed that even mild glycaemic disorders were associated with increased perinatal risk and adverse pregnancy outcomes [1]. Thus, the GDM diagnostic criteria were amended by the International Association of the Diabetes and Pregnancy Study Groups (IADPSG) and adopted by the World Health Organization (WHO [2]). This amendment rendered a single abnormal OGTT value sufficient to diagnose GDM, increasing GDM incidence from 4–5–7–10% and emphasizing the potential contribution of every value to adverse pregnancy outcomes [3]. The HAPO study and recent studies, like TOBOGM (Treatment of Gestational Diabetes Mellitus Diagnosed Early in Pregnancy), have highlighted the significance of prompt GDM detection and the need for early intervention to prevent complications [4, 5].

GDM has been associated with several pregnancy complications, such as prematurity, macrosomia, large-for-gestational-age (LGA) neonates, low Apgar score, pre-eclampsia, cesarean delivery, shoulder dystocia, perinatal injury, neonatal hypoglycemia, and neonatal intensive care unit (NICU) admission. Moreover, women with GDM history have a nearly 10-fold increased risk of developing type 2 diabetes in the future. In contrast, the offspring have a higher risk of childhood insulin resistance and glycaemic disorders [6, 7]. Results from several studies support the hypothesis that abnormal OGTT results could indicate different GDM subtypes which subsequently may be associated with distinct adverse pregnancy outcomes [8–10]. Fasting hyperglycemia indicates primarily hepatic insulin resistance (impaired insulin sensitivity), while post-load hyperglycemia indicates defective insulin secretion and insulin resistance at the skeletal muscle level [11, 12]. These mechanisms have been confirmed as part of the glycaemic physiology of pregnancy [13].

The present study investigated whether OGTT positivity at different time points impacts perinatal outcomes in pregnancies complicated by GDM. It is the first study to evaluate this association in a Greek population.

## Materials and methods

A retrospective cohort study was conducted, including pregnant women with GDM who were managed and gave birth from January 2020 to December 2023 at Alexandra Hospital, a referral tertiary perinatal center in Athens, Greece. In Greece, a screening OGTT is performed on every pregnant woman without known pre-existing diabetes. Data were extracted from the Diabetes Centre medical records and the Department of Obstetrics birth records. GDM diagnosis was based on the IADPSG criteria using a 2 h 75 g OGTT performed between the 24 and 28th gestational week. The diagnostic glucose concentrations for GDM were ≥92 mg/dl (5.1 mmol/l), ≥180 mg/dl (10 mmol/l), and ≥153 mg/dl (8.5 mmol/l) at 0, 60, and 120 min time points, respectively. At least one abnormal value at any time point confirmed GDM diagnosis.

Missing OGTT or perinatal data (34 women) and pre-existing diabetes (39 women) were the exclusion criteria. Women were classified into three groups according to abnormal OGTT concentrations: isolated fasting hyperglycemia (group A), isolated post-load hyperglycemia (group B), and combined hyperglycemia (group C). The primary obstetric outcomes were prematurity and cesarean section (primary or emergency), and the neonatal outcomes included macrosomia, large-for-gestational-age (LGA) and small-for-gestational-age (SGA) neonates, 1-min Apgar score, presence of congenital malformations, and NICU admission. Macrosomia was defined as birth weight ≥4000 g, LGA as birth weight ≥90th percentile for gestational age, SGA as birth weight ≤10th percentile for gestational age, and prematurity as delivery before the 37th gestational week.

The extracted maternal and neonatal characteristics included maternal age, maternal race (Caucasian, Asian, or Black), body mass index (BMI) before pregnancy, gestational week during OGTT performance, glycated hemoglobin (HbA_1C_) level at the time of OGTT, family history of diabetes mellitus, insulin therapy, hypertensive disorders of pregnancy, conception by Assisted Reproductive Technologies (ART), maternal smoking during pregnancy, presence of polycystic ovary syndrome (PCOS), perinatal injury, neonatal sex, and birth weight. All women performed lifestyle modifications (aerobic exercise at least 30 min/day), in the absence of obstetric contraindications, and received medical nutrition therapy (MNT) including at least 1800 kcal/day (35–45% low-glycemic index carbohydrates, 20–25% protein, and 30–40% fat). Women were asked to self-monitor their blood glucose concentrations using finger-prick testing at least four times per day (morning fasting, 1 h after breakfast, 1 h after lunch, and 1 h after dinner). The criteria for insulin therapy initiation included persistently high fasting or postprandial glucose concentrations (>95 mg/dl or >140 mg/dl, respectively) despite MNT and exercise, asymmetric fetal macrosomia (determined by fetal abdominal circumference >75th percentile), and polyhydramnios. Oral hypoglycemic agents (metformin or glyburide) were not administered to any woman, as they are not recommended for GDM management by the national clinical practice guidelines followed in our center [14, 15].

The study was carried out in compliance to the Declaration of Helsinki for conducting clinical research involving human subjects. All participants provided informed consent to participate in the present study. The study conduction was approved by the Institutional Ethics Committee of Alexandra Hospital, Athens, Greece (reference number: 3/22-03-2023).

### Statistical analysis

Data were expressed as mean ± standard deviation (SD) for continuous variables or as absolute numbers and percentages in parentheses for categorical variables. Multivariable linear (for the outcome: 1-min Apgar score) and logistic regression analyses (for the outcomes: prematurity, primary or emergency cesarean section, macrosomia, congenital malformations, and NICU admission) were performed, and the results were expressed as betas for linear or odds ratios (OR) for logistic regression analyses with a 95% confidence interval (95% CI). The level of significance was set at 5%. The statistical analyses were performed using R (version 4.4.1). Maternal age, BMI, HbA_1C_ at the time of OGTT, family history of diabetes mellitus, insulin therapy, hypertensive disorders of pregnancy, and conception by ART were assessed as potential confounders.

## Results

**Fig. 1:**
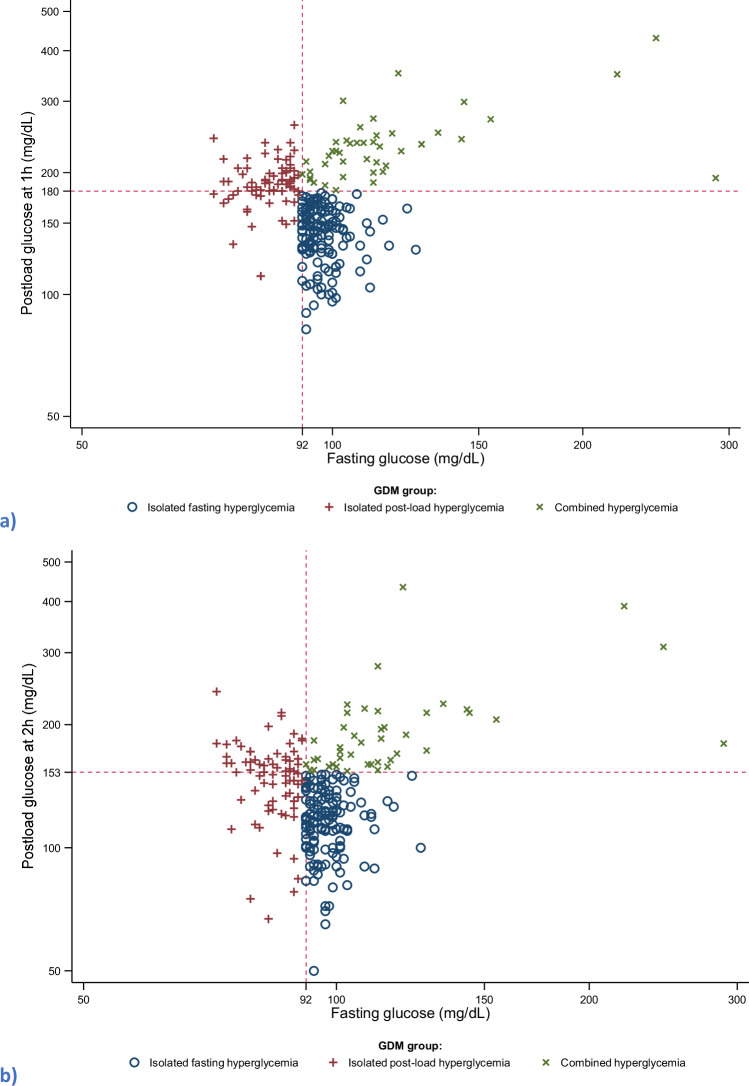
Scatter plots of OGTT concentrations distribution at 1 h (**a**) and 2 h (**b**) among GDM groups. The dotted lines represent the threshold glucose concentrations for GDM diagnosis (IADPSG criteria). Each participating woman is represented with a unique mark. GDM gestational diabetes mellitus, IADPSG international association of the diabetes and pregnancy study groups, OGTT oral glucose tolerance test

**Fig. 2:**
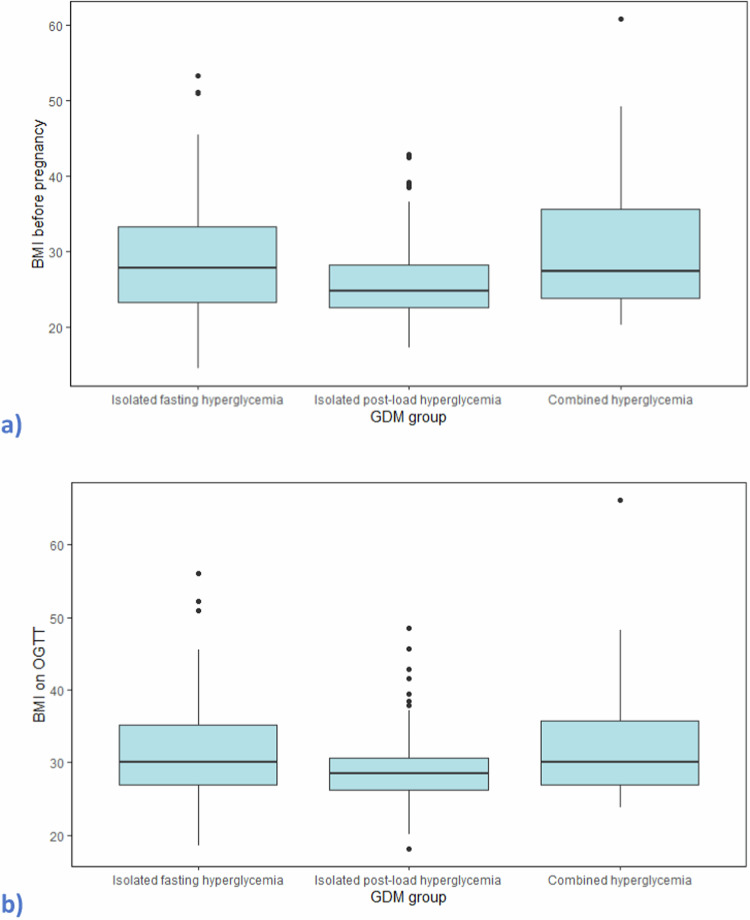
Boxplots of maternal BMI before pregnancy (**a**) and during OGTT (**b**) among GDM groups. BMI body mass index, GDM gestational diabetes mellitus, OGTT oral glucose tolerance test

A total of 257 women were considered eligible for inclusion. Groups A, B, and C included 139 (54.1%), 75 (29.2%), and 43 (16.7%) women, respectively, indicating fasting hyperglycaemia as the most common GDM subtype in our cohort. Descriptive characteristics and pregnancy outcomes per group are summarised in Table 1. The mean maternal age was 34.1 ± 6.6 years, the mean gestational age at the OGTT was 26 ± 4.3 weeks, and the BMI on the OGTT was 30.9 ± 6.8 kg/m^2^. In terms of BMI classes distribution across groups, in group A, 73 (52.5%) women were obese, 41 (29.5%) were overweight, 25 (18%) had normal weight, and none was underweight. In group B, 27 (36%) women were obese, 34 (45.3%) were overweight, 13 (17.3%) had normal weight, and 1 (1.4%) was underweight. In group C, 23 (53.5%) women were obese, 15 (34.9%) were overweight, 5 (11.6%) had normal weight, and none was underweight. The distribution of OGTT glucose concentrations is illustrated in Fig. 1. Women in group C had a higher maternal age (mean [SD] 34.9 [5.9] yrs) and a higher BMI before pregnancy and on OGTT (30.6 [9.3] and 32.8 [8.4], respectively) compared with women in the two other groups (Fig. 2).

**Table 1:** Descriptive characteristics and pregnancy outcomes

	Group A	Group B	Group C	Total	
N (%)	139 (54.1)	75 (29.2)	43 (16.7)	257 (100.0)	*p* value
Maternal age (years)	33.5 (7.1)	34.7 (5.9)	34.9 (5.9)	34.1 (6.6)	0.290
Neonatal sex					
Male	82 (56.9)	31 (39.2)	26 (59.1)	139 (52.1)	
Female	62 (43.1)	48 (60.8)	18 (40.9)	128 (47.9)	0.024
BMI before pregnancy (kg/m^2^)	29.0 (7.6)	26.3 (6.2)	30.6 (9.3)	28.4 (7.6)	0.014
BMI on OGTT (kg/m^2^)	31.2 (6.7)	29.2 (5.7)	32.8 (8.4)	30.9 (6.8)	0.017
BMI change (kg/m^2^)	2.5 (2.6)	3.0 (2.3)	2.8 (3.0)	2.7 (2.6)	0.528
Gestational week at OGTT	26.0 (4.5)	26.6 (3.6)	24.9 (4.7)	26.0 (4.3)	0.099
HbA_1C_ on OGTT (%) (mmol/l)	5.2 (0.4) 33	5.0 (0.3) 31	5.3 (0.3) 34	5.2 (0.4) 33	<0.001
Maternal age ≥35 years					
No	69 (49.6)	30 (40.0)	18 (41.9)	117 (45.5)	
Yes	70 (50.4)	45 (60.0)	25 (58.1)	140 (54.5)	0.349
Race					
Caucasian	105 (75.5)	71 (94.7)	35 (81.4)	211 (82.1)	
Asian	18 (12.9)	2 (2.7)	7 (16.3)	27 (10.5)	
Black	16 (11.5)	2 (2.7)	1 (2.3)	19 (7.4)	0.004
ART					
No	133 (95.7)	73 (97.3)	42 (97.7)	248 (96.5)	
Yes	6 (4.3)	2 (2.7)	1 (2.3)	9 (3.5)	0.739
PCOS					
No	133 (95.7)	70 (93.3)	41 (95.3)	244 (94.9)	
Yes	6 (4.3)	5 (6.7)	2 (4.7)	13 (5.1)	0.749
Family history of DM					
No	100 (71.9)	54 (72.0)	30 (69.8)	184 (71.6)	
Yes	39 (28.1)	21 (28.0)	13 (30.2)	73 (28.4)	0.958
Insulin therapy					
No	26 (18.7)	38 (50.7)	5 (11.6)	69 (26.8)	
Yes	113 (81.3)	37 (49.3)	38 (88.4)	188 (73.2)	<0.001
Smoking during pregnancy					
No	121 (87.1)	65 (86.7)	33 (76.7)	219 (85.2)	
Yes	18 (12.9)	10 (13.3)	10 (23.3)	38 (14.8)	0.229
Gestational hypertension					
No	127 (91.4)	71 (94.7)	38 (88.4)	236 (91.8)	
Yes	12 (8.6)	4 (5.3)	5 (11.6)	21 (8.2)	0.466
Pre-eclampsia					
No	134 (98.5)	71 (97.3)	43 (100.0)	248 (98.4)	
Yes	2 (1.5)	2 (2.7)	0 (0.0)	4 (1.6)	0.515
Apgar score (1-min)	8 (1)	8 (1)	8 (1)	8 (1)	0.426
Birth weight (g)	3221 (525)	3030 (512)	3208 (591)	3163 (538)	0.039
Primary cesarean section					
No	47 (33.8)	26 (34.7)	12 (27.9)	85 (33.1)	
Yes	92 (66.2)	49 (65.3)	31 (72.1)	172 (66.9)	0.727
Urgent cesarean section					
No	121 (87.1)	72 (96.0)	39 (90.7)	232 (90.3)	
Yes	18 (12.9)	3 (4.0)	4 (9.3)	25 (9.7)	0.108
Prematurity					
No	108 (77.7)	60 (80.0)	30 (69.8)	198 (77.0)	
Yes	31 (22.3)	15 (20.0)	13 (30.2)	59 (23.0)	0.429
Macrosomia					
No	129 (92.8)	74 (98.7)	40 (93.0)	243 (94.6)	
Yes	10 (7.2)	1 (1.3)	3 (7.0)	14 (5.4)	0.175
LGA					
No	123 (88.5)	73 (97.3)	36 (83.7)	232 (90.3)	
Yes	16 (11.5)	2 (2.7)	7 (16.3)	25 (9.7)	0.032
SGA					
No	131 (94.2)	69 (92.0)	42 (97.7)	242 (94.2)	
Yes	8 (5.8)	6 (8.0)	1 (2.3)	15 (5.8)	0.448
Congenital malformations					
No	137 (98.6)	74 (98.7)	43 (100.0)	254 (98.8)	
Yes	2 (1.4)	1 (1.3)	0 (0.0)	3 (1.2)	0.725
NICU admission					
No	125 (89.9)	70 (93.3)	40 (93.0)	235 (91.4)	
Yes	14 (10.1)	5 (6.7)	3 (7.0)	22 (8.6)	0.642

Data are expressed as mean ± standard deviation (SD) for continuous variables and n (%) for categorical variables. Group A: isolated fasting hyperglycemia; Group B: isolated post-load hyperglycemia; Group C: combined hyperglycemia (group C)

*ART* assisted reproductive technologies, *BMI* body mass index, *HbA*_*1C*_ glycated hemoglobin, *LGA* large-for-gestational-age, *NICU* neonatal intensive care unit, *OGTT* oral glucose tolerance test, *PCOS* polycystic ovary syndrome, *SGA* small-for-gestational-age

**Fig. 3:**
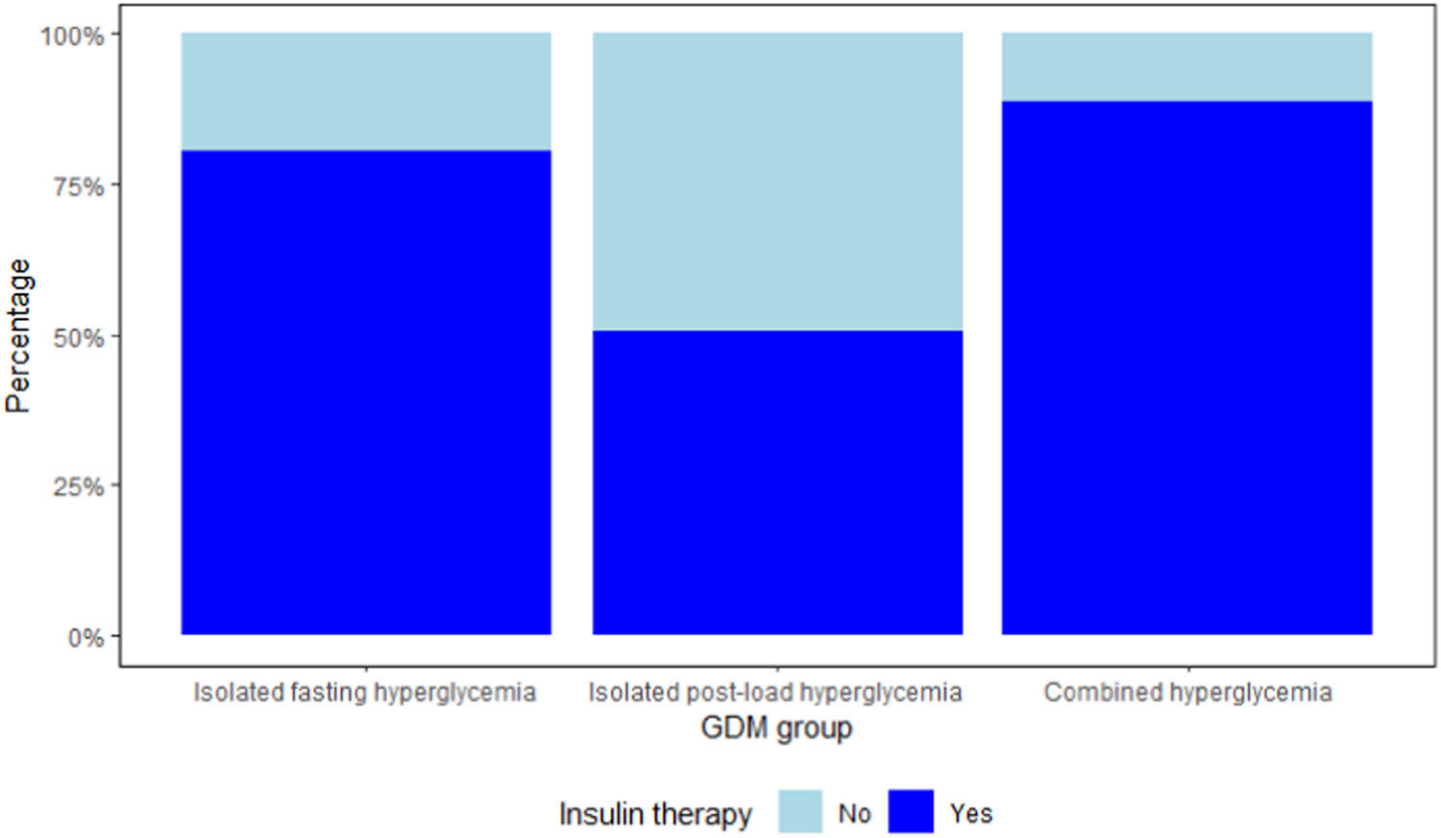
Barplot of insulin therapy among the study groups

Regarding ethnic differences, the representation of women of Asian descent was higher in groups A and C compared with group B (*p* = 0.004). Insulin therapy was required in 188 (73.2%) women. Women with fasting or combined hyperglycemia (groups A and C) were more likely to receive insulin therapy compared to group B (88.4 and 81.3% vs. 49.3%, *p* < 0.001) (Fig. 3). Additionally, women in groups A and C had higher HbA_1C_ concentrations at the time of OGTT compared with group B (5.2 and 5.3% vs 5%, *p* = 0.001), while their neonates had higher birth weight (3221 ± 525 g and 3208 ± 512 g vs. 3030 ± 591 g, *p* = 0.039) and an increased risk of being LGA compared to those in group B (11.5 and 16.3% vs. 2.7%, *p* = 0.032). Congenital malformations were detected only in three neonates; one of them presented a cardiac defect, and two had limb abnormalities (hexadactyly and clubfoot). The three women shared the feature of severe (class II) obesity (BMI > 35 kg/m^2^).

Regarding the multivariable analyses evaluating the perinatal outcomes between studied groups, group A presented higher risk of prematurity (OR 1.28, 95% CI (0.58–2.81), *p* = 0.54) and congenital malformations (OR 7.19, 95% CI 0.19–264, *p* = 0.28). Group B presented lower risk of macrosomia (OR 0.25, 95% CI (0.03–2.16), *p* = 0.21) and cesarean section (OR 0.79, 95% CI 0.39–1.58, *p* = 0.50). However, these results were not statistically significant. A potential explanation may be the relatively small number of cases included in several of the multivariable analyses [e.g. congenital anomalies (3 cases)]. Limited sample size can reduce the statistical power of multivariable models to detect associations.

## Discussion

The present study aimed to determine the association between abnormal glucose concentrations during the OGTT and adverse pregnancy outcomes in women with GDM. The results confirmed that GDM is a heterogeneous condition characterized by various subtypes, each defined by distinct metabolic phenotypes and pregnancy outcomes. They support the view that impaired insulin sensitivity and/or insulin secretion are key factors contributing to GDM pathophysiology, a finding consistent with previous literature [16, 17]. The high proportion of women requiring insulin therapy in our cohort, above the typical 15–30% range, reflects the higher prevalence of high-risk pregnancies managed at our referral tertiary hospital, where optimal care is provided for potential perinatal complications [18].

In the present cohort, the group with isolated fasting hyperglycemia showed higher HbA_1C_ levels, an increased need for insulin therapy, higher neonatal birth weight, and an elevated risk for LGA neonates, aligning with relevant original studies and a meta-analysis [19–22]. Isolated fasting hyperglycemia has been linked to increased insulin resistance, maternal obesity, increased need for additional insulin therapy, risk of neonatal macrosomia, and poor glycemic control [23, 24]. Advanced maternal age, higher BMI, and higher glucose concentrations in the OGTT have been considered predictors of glucose-lowering medication requirements during pregnancy [25].

On the other hand, isolated post-load hyperglycemia indicates impaired glucose tolerance (IGT) and has been associated with defects in the early and late phases of insulin secretion [26]. In the present study, this group was associated with the most favorable metabolic phenotype regarding maternal and neonatal outcomes. Relevant studies also indicate a milder metabolic dysregulation in women with isolated post-load hyperglycemia, which is similar to impaired glucose tolerance observed in non-pregnant women [27]. Interestingly, comparable insulin sensitivity between women with isolated post-load hyperglycemia and women with normoglycaemia has been reported [28]. A relevant study involving 1,813 women according to their OGTT concentrations and degree of insulin resistance, as measured by the Matsuda index, showed that women with GDM and low insulin resistance had similar phenotypes and pregnancy outcomes with normoglycemic women [29, 30]. However, it is important to note that postpartum risk of pre-diabetes and type 2 diabetes was consistent across different GDM subtypes, emphasizing that all women with a history of GDM are at higher risk, independent of their OGTT results [31].

Additionally, women with combined hyperglycemia exhibited mixed metabolic dysregulation and a higher incidence of insulin use, similar to previous reports that noted increased insulin resistance and higher risks of perinatal complications in this subgroup [32]. In this study, the group with combined hyperglycemia (group C) shared characteristics with the group of isolated fasting hyperglycemia (group A) in terms of higher maternal BMI, gestational weight gain, and HbA_1C_, and higher neonatal birth weight, underlining a consistent pattern of adverse pregnancy outcomes seen in these subtypes. The high prevalence of obesity in groups A and C implies a predisposition to metabolic syndrome development. Research on GDM subtypes has indicated that women with combined hyperglycemia have an increased risk of developing type 2 diabetes postpartum, which warrants long-term follow-up [33, 34]. These findings may point towards considering the fasting and combined hyperglycemia groups in pregnancy as high-risk.

In terms of ethnic differences, the high representation of women of Asian descent in the groups bearing the highest metabolic risk in the present cohort follows the literature [35]. GDM prevalence is disproportionately higher in women from South Asia (India, Pakistan, Sri Lanka, etc.) and South East Asia (Vietnam, Thailand, Malaysia, Philippines) compared to women of Caucasian origin [36]. Foreign-born women present a higher risk of GDM development compared to those who were born in the country having the OGTT [37, 38]. In our cohort, almost all women were born and raised in their original countries. GDM management in this diverse population can be challenging due to ethnic differences in food choices and eating patterns attributed to culture and religion [39, 40]. Thus, clinical awareness should be raised for early GDM detection and management in women with a high-risk ethnic background. An individualized dietary plan is usually offered to ensure optimal compliance with MNT.

The present study presents some strengths and certain limitations. First, it is the only study evaluating the association of abnormal OGTT concentrations with perinatal outcomes in pregnancies complicated by GDM in Greece. Second, the sample size is sufficiently large given the Greek population and the relatively low annual birth rate (<7 births/1000 people) [41]. The lack of insulin data (insulin sensitivity and insulin secretion) during the OGTT constitutes a major limitation of this study, which is attributed to its retrospective design. Other limitations include the single-center setting, the absence of a control group of normoglycemic women, and the relatively small number of cases included in the outcome-specific multivariable analyses which may have limited the power to detect associations.

Regarding clinical implications, the present study is important as it recognized parameters indicating unfavorable metabolic phenotypes that can be used to guide tailored treatment in GDM. An OGTT result indicating fasting or combined hyperglycemia will raise clinical awareness towards a high risk of need for glucose-lowering medications, neonatal macrosomia, or LGA newborns. Consequently, women will be closely monitored to receive early intervention if needed.

In conclusion, the present study highlights the importance of GDM classification into subtypes according to the OGTT results to guide treatment. The different GDM subtypes differ in terms of therapeutic management and prognosis. Compared with isolated post-load hyperglycemia in the OGTT, isolated fasting hyperglycemia or combined hyperglycemia were associated with less favorable pregnancy outcomes. Further prospective studies should evaluate the pathophysiological heterogeneity between the GDM subtypes and determine whether the OGTT results could individualize GDM management by applying personalized treatment strategies.

## Data Availability

The data supporting the findings of this study are available from the corresponding author upon reasonable request.
